# Genome Sequence of *Rhodoferax antarcticus* ANT.BR^T^; A Psychrophilic Purple Nonsulfur Bacterium from an Antarctic Microbial Mat

**DOI:** 10.3390/microorganisms5010008

**Published:** 2017-02-21

**Authors:** Jennifer M. Baker, Carli J. Riester, Blair M. Skinner, Austin W. Newell, Wesley D. Swingley, Michael T. Madigan, Deborah O. Jung, Marie Asao, Min Chen, Patrick C. Loughlin, Hao Pan, Yuankui Lin, Yaqiong Li, Jacob Shaw, Mindy Prado, Chris Sherman, Joseph Kuo-Hsiang Tang, Robert E. Blankenship, Tingting Zhao, Jeffrey W. Touchman, W. Matthew Sattley

**Affiliations:** 1 Division of Natural Sciences, Indiana Wesleyan University, Marion, IN 46953, USA; jenn.baker@myemail.indwes.edu (J.M.B.); carliriester@gmail.com (C.J.R.); blair.skinner@myemail.indwes.edu (B.M.S.); austinwayne.newell@gmail.com (A.W.N.);; 2 Department of Biological Sciences, Northern Illinois University, DeKalb, IL 60115, USA; wswingley@niu.edu; 3 Department of Microbiology, Southern Illinois University, Carbondale, IL 62901, USA; madigan@siu.edu (M.T.M.); debjung@siu.edu (D.O.J.); 4 Department of Microbiology, The Ohio State University, Columbus, OH 43210, USA; marie.asao@gmail.com; 5 School of Life and Environmental Sciences, The University of Sydney, New South Wales 2006, Australia; min.chen@sydney.edu.au (M.C.); patftct@gmail.com (P.C.L.); dylanwind@gmail.com (H.P.); ylin2801@uni.sydney.edu.au (Yu.L.); Yaqiong.li@sydney.edu.au (Ya.L.); 6 Departments of Biology and Chemistry, Washington University in Saint Louis, St. Louis, MO 63130, USA; jacobsshaw@gmail.com (J.S.); mcpty5@gmail.com (M.P.); shermanc@wustl.edu (C.S.); Joseph.Tang.ctr@us.af.mil (J.K.-H.T.); blankenship@wustl.edu (R.E.B.); 7 School of Life Sciences, Arizona State University, Tempe, AZ 85287, USA; tgtg.zhao@gmail.com (T.Z.); jeffrey.touchman@monsanto.com (J.W.T.)

**Keywords:** purple anoxygenic phototroph, photosynthesis gene cluster, light-harvesting complex, psychrophile, Antarctica, *Rhodoferax antarcticus*, nitrogen fixation, nitrogenase

## Abstract

*Rhodoferax antarcticus* is an Antarctic purple nonsulfur bacterium and the only characterized anoxygenic phototroph that grows best below 20 °C. We present here a high-quality draft genome of *Rfx. antarcticus* strain ANT.BR^T^, isolated from an Antarctic microbial mat. The circular chromosome (3.8 Mbp) of *Rfx. antarcticus* has a 59.1% guanine + cytosine (GC) content and contains 4036 open reading frames. In addition, the bacterium contains a sizable plasmid (198.6 kbp, 48.4% GC with 226 open reading frames) that comprises about 5% of the total genetic content. Surprisingly, genes encoding light-harvesting complexes 1 and 3 (LH1 and LH3), but not light-harvesting complex 2 (LH2), were identified in the photosynthesis gene cluster of the *Rfx. antarcticus* genome, a feature that is unique among purple phototrophs. Consistent with physiological studies that showed a strong capacity for nitrogen fixation in *Rfx. antarcticus*, a nitrogen fixation gene cluster encoding a molybdenum-type nitrogenase was present, but no alternative nitrogenases were identified despite the cold-active phenotype of this phototroph. Genes encoding two forms of ribulose 1,5-bisphosphate carboxylase/oxygenase were present in the *Rfx. antarcticus* genome, a feature that likely provides autotrophic flexibility under varying environmental conditions. Lastly, genes for assembly of both type IV pili and flagella are present, with the latter showing an unusual degree of clustering. This report represents the first genomic analysis of a psychrophilic anoxygenic phototroph and provides a glimpse of the genetic basis for maintaining a phototrophic lifestyle in a permanently cold, yet highly variable, environment.

## 1. Introduction

Anoxygenic phototrophic bacteria are widespread in nature, and within this group, the purple nonsulfur bacteria (PNB) are by far the most metabolically diverse. Species of PNB are either *Alpha*- or *Betaproteobacteria* and are noted for their capacity to grow both phototrophically (anoxic/light) and chemotrophically (oxic/dark). This broad metabolic diversity allows PNBs to adjust their metabolism to fit available conditions and resources in a wide variety of habitats [1].

**Figure 1:**
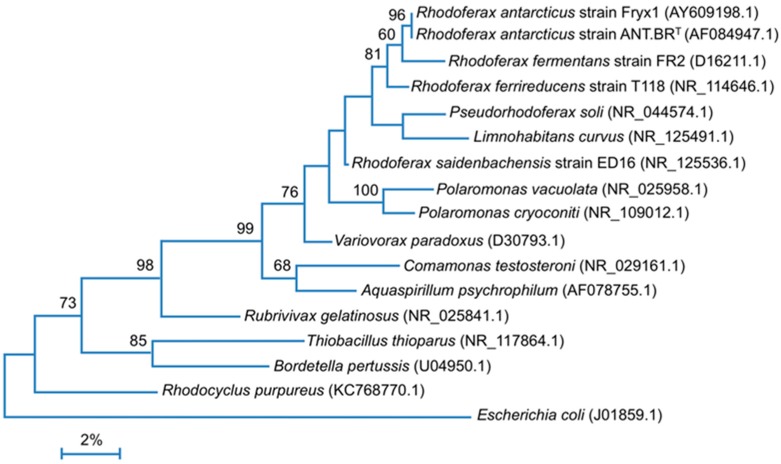
The 16S rRNA gene phylogenetic tree of *Rfx*. *antarcticus* strain ANT.BR^T^ and related *Betaproteobacteria* with *Escherichia coli* (*Gammaproteobacteria*) as the outgroup. rRNA gene sequences were obtained from the National Center for Biotechnology Information (NCBI) GenBank database [6] and aligned with ClustalW using Mega7 [7], with a final data set of 1380 nucleotides. Phylogenetic analysis was conducted using the maximum likelihood method in conjunction with the Jukes–Cantor correction [8]. Bootstrap values above 50 (100 replicates) are shown at their respective nodes.

PNBs have been isolated from several extreme environments, including hot, cold, acidic, alkaline, and hypersaline [1,2]. The success of PNBs in these harsh habitats infers that they have evolved important biochemical modifications to support photosynthesis under stressful conditions. *Rhodoferax antarcticus*, one of four species of the genus and a member of the *Betaproteobacteria* (Figure 1), is the first purple bacterium isolated from a permanently cold environment, a microbial mat on Ross Island, McMurdo, Antarctica [3] (Table 1). The organism is a small curved rod, highly motile by flagellar means, and contains bacteriochlorophyll (Bchl) *a*. *Rfx. antarcticus* grows at 0 °C and optimally near 15 °C, major tenets of psychrophiles [4]. A second and phenotypically distinct strain of *Rfx. antarcticus*, strain Fryx1 (Figure 1), was isolated from the water column of the permanently ice-covered Lake Fryxell, McMurdo Dry Valleys [5].

Genome sequences are available for several PNBs [9]. However, no genome sequence has been available for a purple bacterium that thrives in constantly cold conditions. The genetic blueprint of such an organism could begin to reveal how photocomplexes and related photosynthetic machinery are altered to function optimally in the cold. To explore these questions, we report here an analysis of the genome sequence of *Rfx. antarcticus* strain ANT.BR^T^. Our results focus on four functional gene sets where this organism shows genomic peculiarities compared with the genomes of PNBs that thrive in temperate environments, peculiarities that may have relevance for the ecology of this Antarctic phototroph.

## 2. Materials and Methods

*Rhodoferax antarcticus* strain ANT.BR^T^ was isolated from a microbial mat in a pond near Cape Royds, Ross Island, Antarctica [3] (Table 1). A single colony was grown anaerobically and total DNA was isolated using proteinase K treatment followed by phenol extraction. Genome sequencing was performed using a random shotgun approach. Sequence reads were generated with three technologies to improve quality, aid assembly, and correct for systematic error introduced by any single method. Nearly 65 million paired-end reads were generated on the Illumina HiSeq platform resulting in an estimated 1539-fold sequence coverage of the chromosome (~3.8 million base pairs) and 2847-fold coverage of the single plasmid (198,615 base pairs) (Table 2). Additionally, 371,330 random reads representing 33-fold sequence coverage were generated by pyrosequencing on a Roche-454 GS20 sequencer (Hoffman-La Roche AG, Basel, Switzerland). Finally, 11,426 paired-end reads representing 1.5-fold sequence coverage were generated from a large insert fosmid library in the pEpiFOS-5 vector (insert sizes ranging from 28–47 kb) using dye terminator chemistry on an ABI 3730xl automated sequencer (Applied Biosystems, Waltham, MA, USA); these reads were used as a scaffold. The sequences were assembled using Velvet with default settings [10].

Automated annotation of the genome was performed using the University of Maryland School of Medicine Institute for Genome Science’s Prokaryotic Annotation Pipeline [11] within the Analysis Engine service [12]. Pairwise alignments were generated using BLAST-extend-repraze (BER) [13], which employs a combination of BLAST and Smith–Waterman algorithms. In addition, the process includes gene identification with Glimmer, Hidden Markov Model (HMM) searches, transmembrane (Tm) HMM searches, SignalP predictions, and automatic annotations from AutoAnnotate. Additionally, the annotation tool Manatee [14] was used to manually review and confirm the annotation of every gene. Pseudogenes contained one or more mutations that would ablate expression; each inactivating mutation was subsequently checked against the original sequencing data.

In addition to Manatee, statistics in Table 2 were generated using the Pfam database (v. 30.0) [15], the SignalP database (v. 4.1) [16], the TMHMM database (v. 2.0) [17], and CRISPRFinder (v. 2.0) [18]. For the method of phylogenetic tree assembly, see the legend to Figure 1. This Whole Genome Shotgun project has been deposited at DDBJ/EMBL/GenBank under the accession MSYM00000000. The version described in this paper is version MSYM01000000.

## 3. Results and Discussion

### 3.1. Genome Properties

The genome of *Rhodoferax antarcticus* ANT.BR^T^ consists of a single circular chromosome of approximately (due to the draft nature of the sequence) 3,809,266 base pairs with a G+C content of 59.1%. *Rfx. antarcticus* also possesses a sizeable plasmid (198,615 bp) with a significantly different G+C content than that of the chromosome (Table 2). Of the 4324 genes identified in the total genome, 4257 were protein-encoding genes, 67 were ribosomal or transfer RNAs, and 228 were putative pseudogenes. Most of the pseudogenes were putative transposases and hypothetical proteins rather than proteins having key metabolic or physiological functions (e.g., none were assigned roles relating to phototrophic energy conservation). A putative function and role category was assigned to 65.8% of protein-encoding genes, while the remaining genes were annotated as hypothetical, conserved hypothetical, or as genes of unknown function (Table 3).

### 3.2. Major Photosynthesis Genes

**Figure 2:**
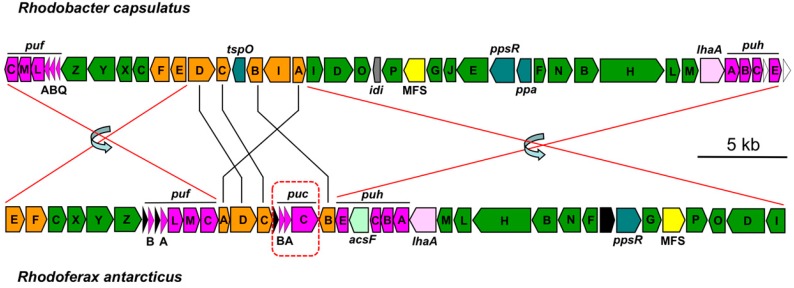
Superoperonal photosynthetic gene cluster of *Rfx. antarcticus* ANT.BR^T^. The *puc* genes, which are absent from *Rhodobacter capsulatus*, are present in the *Rfx*. *antarcticus* cluster. Lines indicate rearrangements in gene synteny and arrows indicate inversions. Color key: green, bacteriochlorophyll synthesis (*bch*); orange, carotenoid synthesis (*crt*); pink, photosynthetic reaction centers (*puh*); light-harvesting complexes (*puf* and *puc*); teal, regulatory proteins; white, uncharacterized proteins; black, hypothetical proteins. All other colors are unique for clarity.

As is typical in purple bacteria, *Rhodoferax antarcticus* contains a photosynthesis gene cluster (PGC) in which most of the photosynthetic genes are arranged within a single superoperon on the circular chromosome (in the case of *Rfx. antarcticus*, at the 2.2 Mb mark). A comparison of gene synteny with that of the PGC from the well-studied species *Rhodobacter* (*Rba*.) *capsulatus* reveals two sizable inversions of superoperonal clusters in *Rfx. antarcticus* that include the *bch*, *puf*, and *puh* genes (Figure 2). *Rfx. antarcticus* contains just one copy of the *pucABC* operon, which encodes the peripheral light-harvesting antenna complex; interestingly, this operon is embedded within several *crt* (carotenoid biosynthesis) genes in the PGC. In all other purple nonsulfur bacteria, including *Rba. capsulatus* (Figure 2), *puc* genes are absent from the PGC and are instead dispersed in other regions of the chromosome, often in multiple copies.

As is the case for many other PNBs, the *Rfx. antarcticus* PGC also contains a gene encoding an unspecified major facilitator superfamily (MFS) protein (Figure 2) nested between *bchG* (GRFA_2400) and *bchP* (GRFA_2402; Figure 2), genes that encode enzymes that catalyze the final steps of bacteriochlorophyll (Bchl) synthesis. Gene products in this ubiquitous superfamily typically function in membrane transport. Due to its proximity to these genes, the MFS gene product may play a key role in the assembly of photosynthetic complexes [19], possibly by facilitating the insertion of Bchl *a* into membranes. It is worth noting that intracytoplasmic membranes typical of purple bacteria were not observed in electron micrographs of low-light grown cells of *Rfx. antarcticus* [3]. Some purple bacteria, such as *Rhodocyclus* species and *Rubrivivax gelatinosus*—purple bacteria closely related to *Rfx. antarcticus* (Figure 1)—have minimal intracytoplasmic membrane systems and rely on extensions of the cytoplasmic membrane to house the photosynthetic machinery [20,21,22]. It may be that *Rfx. antarcticus* is another such example of this.

All genes necessary for Bchl *a* biosynthesis were identified in the *Rfx. antarcticus* genome, although not all are located in the PGC (Figure 2). Apart from the PGC, there are two other regions of the chromosome that contain Bchl biosynthesis genes. The genes *bchJ* (GRFA_3894) and *bchE* (GRFA_3895) are found contiguously at approximately 3.6 Mb, and a second copy of the genes *bchI* (GRFA_1518) and *bchD* (GRFA_1519) are found at approximately 1.4 Mb. BLASTp analyses show moderate to low protein sequence identity (*bchI*- 91% coverage, 45% identity; *bchD*- 68% coverage, 36% identity) and disparate lineages (exclusively *Betaproteobacteria* at 1.4 Mb versus *Beta-*, *Alpha-*, and *Gammaproteobacteria* at 3.6 Mb) between the two sets of *bchID*. The purpose of maintaining two phylogenetically distinct sets of genes that encode the enzyme magnesium chelatase, which adds Mg^2+^ to the tetrapyrrole ring structure of Bchl *a*, in separate regions of the *Rfx. antarcticus* genome is unclear; however, it is possible that they are regulated differently by temperature or some other environmental variable.

Also noteworthy was the presence of *acsF* (GRFA_2386), located within the PGC, and *bchE* (GRFA_3895), which was located on a separate region of the chromosome outside of the PGC. These two genes encode versions of magnesium-protoporphyrin IX monomethyl ester cyclase (EC 1.14.13.81) that should enable *Rfx. antarcticus* to synthesize Bchl *a* under both anaerobic and aerobic conditions, respectively [23,24]. This flexibility emphasizes the metabolic versatility of *Rfx. antarcticus*, an organism that may need to oscillate regularly between aerobic and anaerobic (and chemotrophic or phototrophic) metabolisms, depending on the prevailing physicochemical conditions in its microbial mat habitat. Especially during the winter-to-spring and fall-to-winter transitions in Antarctica, light and O_2_ levels may fluctuate rapidly in such a habitat.

Carotenoids are important accessory pigments that assist phototrophs in collecting light energy and protect the cell from the damaging effects of reactive oxygen species [25,26]. Annotation of the *Rfx. antarcticus* genome revealed the presence of *crt* genes that encode enzymes needed for the complete spheroidene and spirilloxanthin pathways. Whereas most *crt* genes are located in the PGC (Figure 2), *crtI* (GRFA_3896) is located at approximately 3.6 Mb within the same operon as *bchJE*, and a second copy of *crtA* (GRFA_2303) is located at approximately 2.1 Mb. These findings are in agreement with carotenoid analyses of *Rfx. antarcticus*, which showed high levels of hydroxyspheroidene and other spheroidene derivatives and traces of spirilloxanthin and other spirilloxanthin derivatives in this organism [4,19].

### 3.3. Light-Harvesting Complexes

From genomic sequence data, it is evident that *Rfx. antarcticus* can biosynthesize both a core and a peripheral light-harvesting complex. The *puf* operon, which encodes the alpha and beta subunits of the LH1 complex, and the *puc* operon, which encodes the alpha and beta subunits of the peripheral LH2/LH3 complexes, are both located in the PGC of *Rfx. antarcticus* (Figure 2). The presence of genes encoding the LH1 complex is consistent with the absorption spectrum of intact cells given by Madigan et al. [3], which showed an absorbance peak at 866 nm. However, the same spectra failed to yield evidence for an LH2 complex, as no maxima were observed between 850 and 855 nm. However, the presence of absorbance maxima at 819 nm instead suggests that *Rfx. antarcticus* synthesizes an LH3 complex [3]. At least three species of purple bacteria that produce both LH2 and LH3 (*Rhodobacter azotoformans*, *Phaeospirillum molischianum*, and *Rhodoblastus acidophilus*) have been shown to exhibit a change in the ratio of peripheral complexes when light intensity or temperature changes [27,28,29,30]. In these bacteria, low temperatures and/or low light intensity elicit an increase in the expression of LH3 complexes and a decrease in the expression of LH2 complexes. In addition, Mascle-Allemand et al. [28] showed that the transition between spectral forms of peripheral complexes can be complete, and therefore LH2 is not necessary as a mediator between LH3 and LH1.

Spectroscopic data presented by Madigan et al. [3] were gathered from cells grown at 18 °C and low incandescent light. Considering that *Rfx. antarcticus* is capable of growth between 0 and 25 °C, it is reasonable to assume that incubation at 18 °C would have been warm enough to trigger preferential expression of LH2 complexes over LH3 if LH2 complexes could indeed be synthesized. However, no spectral evidence for an LH2 complex was observed, and this suggests that the sole *puc* operon identified in the genome encodes the LH3 complex, responsible for producing absorbance at 819 nm.

The spectrum produced by peripheral light-harvesting complexes depends upon the interactions of the C-3 acetyl group of Bchl *a* with the peripheral complex. If key residues at positions 44 and 45 of the LH2 alpha subunit (PucA) are able to hydrogen bond with the C-3 acetyl group, the spectrum shows a peak near 850 nm, whereas the absence of hydrogen bonding shifts the peak to 820 nm [29,31]. Examination of the amino acid sequence of PucA in *Rfx. antarcticus* revealed two phenylalanine residues at positions 44 and 45, not tyrosine and tryptophan, respectively, which are needed to form hydrogen bonds [29,32]. In addition, protein sequence comparison of the PucA and PucB subunits from *Rfx. antarcticus* to corresponding LH3 sequences from *Phaeospirillum molischianum* [33] revealed conserved motifs, with 38% and 36% sequence identity, respectively.

**Figure 3:**
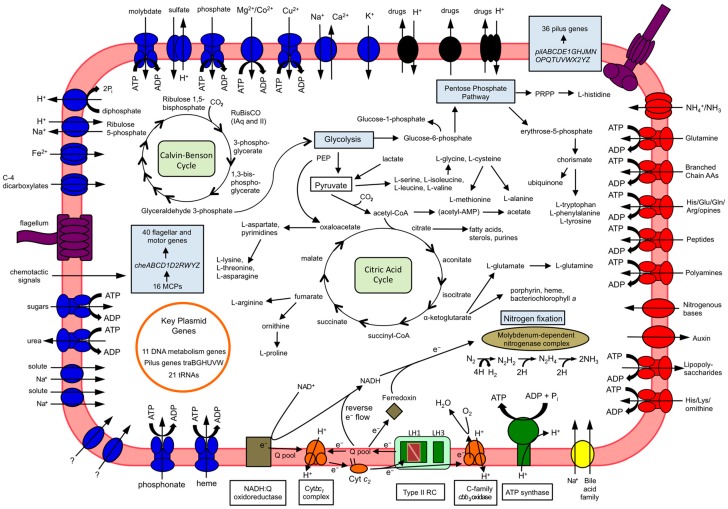
Summary of metabolism and transporters of *Rfx. antarcticus* ANT.BR^T^. Noteworthy features include light-harvesting via a type II (quinone-type) reaction center [25] in association with LH1 and LH3, an intact Calvin–Benson cycle with two forms of RuBisCO, the ability to synthesize 20 amino acids, and antibiotic resistance properties conferred by drug exporters. In addition, both a flagellum and a type IV pilus are shown, providing two means of motility. Interestingly, although biotin is required for growth, no biotin transporter was identified in the genome. However, it is possible that a novel biotin transporter is present among several transporters of undetermined substrates.

These data lead to the hypothesis that, in addition to LH1, *Rfx. antarcticus* produces LH3 (B800/820) complexes to the exclusion of LH2 under all growth conditions, presumably with the arrangement shown in Figure 3. If true, this phenomenon would be unique among purple bacteria. Such an atypical physiological response makes sense considering the habitat of *Rfx. antarcticus*. In the aquatic microbial mat from which strain ANT.BR^T^ was isolated (Table 1), the maximum water temperature during the summer is 8 °C [19,34]. In addition, light levels approach zero during the austral winter, and therefore, in combination with permanently cold temperatures, preferential selection for the ability to synthesize LH3 over LH2 may have occurred in *Rfx. antarcticus*. The production of an LH3 complex to the exclusion of an LH2 complex may be an adaptation that gives *Rfx. antarcticus* a selective advantage in its cold and light-limiting microbial mat habitat.

The LH complexes of *Rfx. antarcticus* are also intriguing considering the spectral differences between the two strains of *Rfx. antarcticus*. Strain Fryx1, which conducts a planktonic lifestyle in the water column of Lake Fryxell, shows an absorbance peak at 836 nm [5]. Fowler et al. [35] demonstrated that a single amino acid substitution (Tyr44 → Phe) in the LH2 alpha subunit primary structure blue-shifted the spectrum to 839 nm, essentially forming a light-harvesting complex having spectral qualities intermediate of LH2 and LH3. Since Fryx1 and ANT.BR^T^ are two strains of the same species, it is possible that, with respect to the alpha44 and 45 positions, Fryx1 has one hydrogen-bond forming residue and one non-hydrogen-bond forming residue, while ANT.BR^T^ maintains two non-hydrogen-bond forming residues. Differences in light quality and availability between the water column of the permanently ice-covered Lake Fryxell and microbial mats of Ross Island ponds could have selected for these differential adaptations in order for these two strains to achieve maximal fitness in their respective environments.

### 3.4. Carbon Metabolism

Due to their metabolic versatility, purple bacteria often inhabit fluctuating environments, which would include the Antarctic microbial mat from which strain ANT.BR^T^ was isolated [2]. Beside the long periods of darkness or light during the respective winter and summer seasons, Antarctic microbial mat communities experience turbulent anoxic/oxic cycles during the seasonal transition periods, stressors for which multiple energy generation pathways and the ability to easily switch between them are beneficial. Like most other purple bacteria, *Rfx. antarcticus* is metabolically versatile, as confirmed by genomic analysis, which revealed genes for both phototrophic (anoxic/light) and heterotrophic (oxic/dark) growth.

Genes encoding all enzymes of the Calvin–Benson cycle are present in the *Rfx. antarcticus* genome (Figure 3). One notable feature is the presence of two forms (IAq and II) of the enzyme ribulose 1,5-bisphosphate carboxylase/oxygenase (RuBisCO), which carboxylates ribulose 1,5-bisphosphate in the first step of the cycle to produce two molecules of 3-phosphoglycerate [25,36]. The presence of multiple RuBisCO forms is thought to confer metabolic versatility since the unique enzymatic properties of each form suit different environmental conditions, such as variations in [CO_2_]/[O_2_] ratios [36]. Presumably, possessing both form IAq (k_cat_ 3.7 s^−1^), which operates best with medium-to-low CO_2_ availability [37], and form II (k_cat_ 5.7 s^−1^), which functions best in high CO_2_ and low O_2_ concentrations, would enable *Rfx. antarcticus* to continue carbon fixation under transitional periods of fluctuating dissolved O_2_ concentrations [36]. In addition, it is possible that the two *Rfx. antarcticus* RuBisCO enzymes differ in their temperature optima for activity.

**Figure 4:**
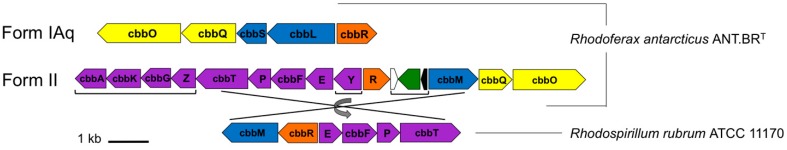
RuBisCO gene cluster arrangements in the purple nonsulfur bacteria *Rfx. antarcticus* and *Rhodospirillum rubrum*. *Rfx. antarcticus* possesses two forms of RuBisCO, forms IAq and II. Brackets indicate the presence of extra genes. Color key: blue, RuBisCO subunits; orange, transcriptional regulators; yellow, RuBisCO activation proteins; purple, CBB cycle metabolic genes; green, cobyrinic acid a,c-diamide synthetase; white, uncharacterized proteins; black, hypothetical proteins.

The form IAq gene cluster arrangement is typical of other purple nonsulfur bacteria, sharing the same synteny as *Rhodospirillum centenum* str. SW (ATCC 51521) [37]. However, the form II gene arrangement in *Rfx. antarcticus* is unusual in that it seems to be a combination of two typical form II gene cluster arrangements. While the *Rfx. antarcticus* form II RuBisCO is directly transcribed with the *cbbQO* genes (GRFA_112 and GRFA_113, respectively), which are linked to post-translational regulation of RuBisCO, the metabolic gene cluster for the remaining components of the Calvin–Benson cycle lies immediately adjacent to and is transcribed in the opposite direction of the transcriptional regulator *cbbR* (GRFA_107; Figure 4). This proximity is unusual considering that Calvin–Benson cycle metabolic genes are normally located elsewhere in the genome when form II RuBisCO and *cbbQO* are transcribed simultaneously [36]. However, bacteria that switch between Calvin–Benson cycle-mediated autotrophy and heterotrophic growth typically have genes for the remaining components of the Calvin–Benson cycle adjacent to the form II RuBisCO [36], suggesting that *Rfx. antarcticus* uses the combination of arrangements to tightly regulate its method of growth depending upon conditions and resources in its environment. Synteny comparisons to the *Rhodospirillum rubrum* form II RuBisCO gene cluster reveals that a segment of the *Rfx. antarcticus* form II cluster has been inverted and includes additional genes (Figure 4). Especially unusual is the presence of three unrelated genes [*cbiA* (GRFA_109), which encodes cobyrinic acid *a*,*c*-diamide synthetase, and two unidentified genes] between *cbbR* and the rest of the gene cluster; their function at this locus is unknown.

The *Rfx. antarcticus* genome also contains complete sets of genes for glycolysis and the citric acid cycle (Figure 3). Genes for the Entner–Doudoroff and pentose phosphate pathways were also identified. Complete catabolic pathways for carbon source utilization were confirmed for all substrates previously shown to support growth of strain ANT.BR^T^, except for fructose. No genes encoding fructokinase, hexokinase, or other enzymes that would facilitate entry of fructose into glycolysis via conversion to another sugar were identified in the genome, despite the fact that fructose supported strong growth of *Rfx. antarcticus* [3]. Also of interest is the presence of genes encoding phosphoenolpyruvate (PEP) carboxylase (EC 4.1.1.31), which adds CO_2_ to PEP to produce oxaloacetate, typically the first step of the C-4 dicarboxylic acid cycle. Since the cycle is presumably incomplete in *Rfx. antarcticus* due to the absence of a gene encoding pyruvate-phosphate dikinase (EC 2.7.9.1), it is postulated that PEP carboxylase and other enzymes of this partial cycle instead facilitate anaplerotic assimilation of CO_2_ [37], thereby replenishing intermediates of the citric acid cycle that have been depleted during amino acid biosynthesis [38]. *Rfx. antarcticus* is also capable of importing C-4-dicarboxylic acids—prime growth substrates for this organism [3]—via a C-4 dicarboxylate ABC transporter; this enables replenishment of citric acid cycle intermediates via a second route and diminishes the requirement for obligatory C-4 dicarboxylate synthesis. A C-family heme-copper *cbb*_3_ oxidase was identified in the *Rfx. antarcticus* genome and presumably serves as the terminal electron acceptor during growth supported by aerobic respiration (Figure 3).

### 3.5. Nitrogen Fixation

**Figure 5:**
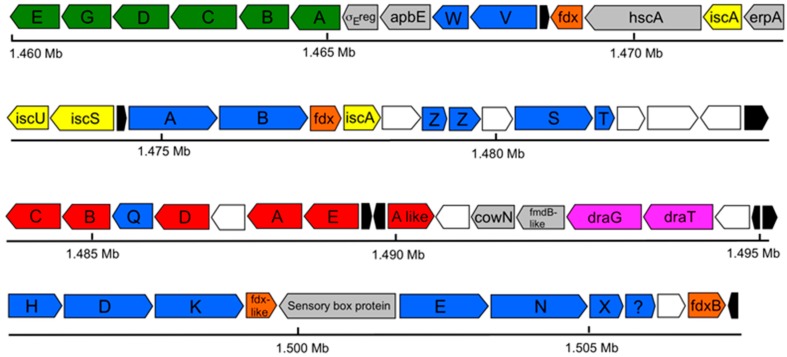
Nitrogen fixation gene cluster of *Rhodoferax antarcticus* ANT.BR^T^. Color key: green, *rnf* genes; blue, *nif* genes; red, *mod* genes; yellow, *isc* genes; pink, *dra* genes; orange, ferredoxin; gray, other nitrogen fixation-associated proteins; white, uncharacterized proteins; black, hypothetical proteins.

Previous observations that *Rfx. antarcticus* can fix N_2_ [3] were supported in the genome, which revealed genes encoding a molybdenum (Mo) nitrogenase. One set of *nif* genes, which encodes Mo-nitrogenase and assembly proteins, as well as one set of *mod* genes, which encodes an ABC transporter for molybdenum, were present in the nitrogen fixation gene cluster (Figure 5). Notably, however, neither *vnf* nor *anf* genes, which encode vanadium (V) and iron (Fe)-only nitrogenases, respectively [39], nor a vanadium transporter, were identified, indicating that the Mo nitrogenase is the only such enzyme present in *Rfx. antarcticus*. Mo-nitrogenases are present in all known diazotrophic *Bacteria* [40], so finding the genes for this enzyme in the *Rfx. antarcticus* genome is not surprising. However, it is surprising that *Rfx. antarcticus* does not have an alternative V- or Fe-only nitrogenase, as V nitrogenases have been shown to have a greater specific activity than Mo nitrogenases at lower temperatures [41], and several other diazotrophic *Proteobacteria* possess alternative nitrogenases [42].

V nitrogenases have been postulated to allow for nitrogen fixation in cold environments [43]. In the absence of such an enzyme, the *Rfx. antarcticus* Mo nitrogenase must be able to function at low temperatures, an uncommon feature among characterized nitrogenases. Acetylene reduction assays by Madigan et al. [3] showed nitrogenase activity down to 2 °C, but no further experiments were done to describe the nitrogenase system of *Rfx. antarcticus.* It might thus be of interest to better characterize the *Rfx. antarcticus nif* gene products to determine what structural features enable this Mo nitrogenase to function under cold conditions.

All *nif* genes absolutely required for nitrogen fixation [44] were present in the *Rfx. antarcticus* genome, but there were some notable absences from the canonical nitrogen fixation cluster. Although *nifJ*, which encodes pyruvate flavodoxin/ferredoxin oxidoreductase, is missing from the *Rfx. antarcticus* nitrogen fixation gene cluster, a gene was identified that is predicted to encode a pyruvate flavodoxin/ferredoxin oxidoreductase (GRFA_1003; EC 1.2.7.1) at approximately 0.9 Mb, adjacent to other electron transfer genes. This suggests that this gene product could serve the function of NifJ. Additionally, while no strong matches for flavodoxin were found in the *Rfx. antarcticus* genome, four different ferredoxin genes are located in the nitrogen fixation cluster, suggesting that ferredoxin, not flavodoxin (*nifF*-encoded or otherwise), may be used by this phototroph to shuttle electrons from the electron transport chain to the *nifH*-encoded Fe protein of the nitrogenase complex [45]. Other *nif* genes missing from the *Rfx. antarcticus* genome include *nifU* (scaffold protein associated with *nifS* activity), *nifM* (Fe protein processing), *nifY* (insertion of FeMo-cofactor into dinitrogenase), and *nifL* (negative regulator of the *nif* genes) [45,46,47]. The composition of *nif* gene clusters varies greatly among species, depending upon the presence or absence of other proteins that can substitute for *nif* gene products [47,48]. For example, both *iscU* (GRFA_1626), a homolog of *nifU*, and two copies of *iscA* (GRFA_1624 and GRFA_2967), which have been postulated to be an alternate scaffold to NifU for the construction of Fe-S clusters [49,50], are present in the *Rfx. antarcticus* nitrogen fixation cluster. The significance of these would require further study.

### 3.6. Motility

**Figure 6:**
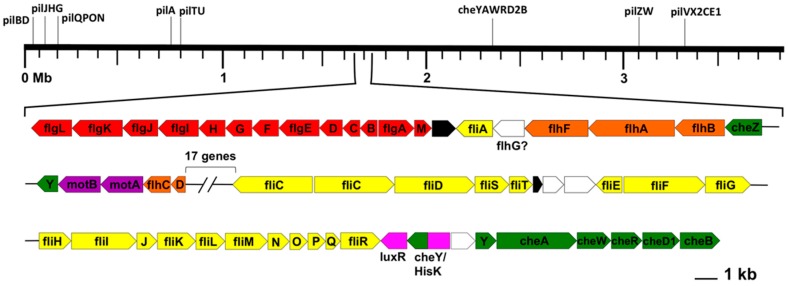
Linear representation of the *Rfx. antarcticus* chromosome, loci of motility genes, and expanded flagellar gene cluster. Type IV pilus genes and a second set of chemotaxis genes (black lines) are distributed down the length of the chromosome, but the flagellar genes are all located in one superoperonal gene cluster. Color key: red, *flg* gene cluster; yellow, *fli* gene cluster; orange, *flh* gene cluster; green, *che* gene cluster; purple, *mot* gene cluster; pink, unrelated characterized proteins; white, uncharacterized proteins; black, hypothetical proteins.

The *Rfx. antarcticus* genome contains genes for motility via flagella and type IV pili, as well as genes for chemotaxis. This genetic evidence supports previous observations by Madigan et al. [3] that cells of *Rfx. antarcticus* are highly motile and possess at least one polar flagellum. However, the arrangement of flagellar genes is unusual in that the genes are located in only two clusters, separated by 17 unrelated genes (Figure 6). Typically, flagellar genes are dispersed over the length of the chromosome, such as in the case of the purple bacterium *Rhodospirillum centenum*, whose five flagellar gene clusters are found at five different loci on the chromosome [37]. In contrast, type IV pilus genes in *Rfx. antarcticus* are scattered in a more typical fashion throughout the chromosome.

Although the components of the flagellar gene cluster of *Rfx. antarcticus* are typical of those of other flagellated bacteria, there are some peculiarities. To date, genomic analyses of cells that synthesize polar flagella have shown that all genes in the *fliEFGHIJKLMNPQR* section of the flagellar gene cluster are transcribed together in the same direction [51]. However, in the *Rfx. antarcticus* flagellar cluster, *fliE* (GRFA_1877) is transcribed in the opposite direction of all other genes in the cluster, which may be the result of an inversion, although the consequence of this change is unclear. In addition, a presumed duplication event has produced a second copy of *fliC*, which encodes flagellin, the protein that forms the filament of the flagellum. The two copies of *fliC* (GRFA_1870 and GRFA_1871), which are adjacent to each other and transcribed in opposite directions, are 90% identical to each other, as determined by BLASTp analysis. In the highly motile, predatory deltaproteobacterium *Bdellovibrio bacteriovorus*, whose genome also encodes multiple copies of flagellin, it was shown that the distinct copies of flagellin form different regions of the flagellum [52]. It is therefore possible that the protein products of the duplicated *fliC* genes also compose different regions of the *Rfx. antarcticus* flagellum.

Although no pili of any type have been observed in cultured *Rfx. antarcticus* cells, it is possible that pili serve an important function for survival in an Antarctic microbial mat. Besides microbial cells, microbial mats also contain debris that provides the required solid surface for twitching motility. This form of motility is pilus-facilitated and involves anchoring the pilus to a solid surface in a grappling hook-type manner and then jerkily reeling the cell toward or across the surface. Within a microbial mat where flagellum-powered swimming may not be practical or possible, twitching motility may be the more efficient form of locomotion.

Unlike the planktonic *Rfx. antarcticus* strain Fryx1, which clearly possesses gas vesicles [5], neither gas vesicles nor the genes that encode them were found in *Rfx. antarcticus* strain ANT.BR^T^. Gas vesicles provide buoyancy and allow cells to change positions in a water column. Although beneficial for a planktonic existence, they would not be useful within the confines of a stratified microbial mat. The mechanisms of motility in these two phylogenetically identical strains therefore seem well suited to their respective environments.

### 3.7. Plasmid Features

The 198.6 kbp plasmid contains 226 open reading frames (ORFs), most of which are annotated as hypothetical or uncharacterized proteins. The G+C content of the plasmid (48.4%) is significantly lower than that of the chromosome (59.1%), which suggests horizontal gene transfer and supports the idea that this replicon is indeed a plasmid rather than a secondary chromosome, as has been described for some other *Beta*- and *Gammaproteobacteria* [53,54]. Extensive analysis of plasmid contents did not identify any unique genes essential to the survival of *Rfx. antarcticus*. However, since the majority of the plasmid genes have undetermined functions, further molecular studies are needed to confirm the accessory nature of this replicon.

Of the plasmid-encoded proteins that have a putative assigned function, 11 participate in DNA metabolism, consistent with analyses by Dziewit and Bartosik [53] that show the highest number of proteins encoded by plasmids from cold-adapted bacteria function in replication, recombination, and repair of DNA. No antibiotic or heavy metal resistance genes were found on the plasmid, although genes for resistance to beta-lactam antibiotics were identified on the chromosome. The plasmid also contains six genes, *traBGHUVW*, associated with conjugation. However, not all genes required for conjugation pilus assembly are present [55], rendering this operon incomplete and leaving *Rfx. antarcticus* likely unable to transfer genes through this mechanism. In addition, no *gvp* gene cluster for gas vesicle formation was identified on the plasmid. Other polar gas-vesiculate bacteria, such as *Octadecabacter arcticus*, contain plasmids having complete *gvp* gene clusters [56]. It is likely that *Rfx. antarcticus* strain Fryx1 [5] gained the capacity to synthesize gas vesicles through lateral transfer of such a *gvp*-containing plasmid.

A final notable feature of the *Rfx. antarcticus* plasmid is the unexpected presence of 21 tRNA genes, constituting about one-third of the tRNA genes in the genome. tRNAscan-SE (v.1.21) [57] analysis reveals that the average Cove score, which indicates the probability that the predicted secondary structure of a tRNA matches the statistical model [58], for the plasmid-encoded tRNAs is significantly lower at 60.6 bits than the average Cove score for the chromosomal tRNAs (79.88 bits). An increasingly higher Cove score indicates an increasingly higher probability that a tRNA is functional once it has been transcribed and assumes its folded conformation [58]. This result brings into question the functionality of the plasmid-encoded tRNAs and indicates that the tRNA genes on this extraneous replicon may be degenerating. Moreover, all tRNA genes on the plasmid duplicate anticodons already present on the chromosome, so it is unclear what function (if any) the plasmid tRNAs serve in translation processes of *Rfx. antarcticus.*

It is possible that the extensive assemblage of potentially degenerate tRNA genes on the plasmid plays a greater role in *Rfx. antarcticus* as a cold-adaptation strategy than as a tool for translation processes. The accumulation of tRNAs for the purpose of cryoprotection has been reported in other psychrophiles [59,60], and it may also be important for *Rfx. antarcticus*. We were unable to identify genes that encode well-known cold-adaptation proteins, including the cold shock proteins CspA or CspB; the cold-adaptive proteins CapA or CapB; ice-nucleating proteins InaQ, InaK, or InaZ; and antifreeze protein AfpA. However, a gene encoding tRNA dihydrouridine synthase A (GRFA_2724), an enzyme that incorporates dihydrouridine into tRNA molecules to increase their conformational flexibility [61], was present in the *Rfx. antarcticus* genome. We also identified genes encoding an ABC-type transporter for glycine betaine, which has been shown to function as a cryoprotective compatible solute in a variety of psychrophilic bacteria [62]. Together, these features may be essential for *Rfx. antarcticus* to maintain viability and sustain growth in permanently cold Antarctic mats.

## 4. Conclusions

As *Rfx*. *antarcticus* strain ANT.BR^T^ is the first psychrophilic anoxygenic phototroph to have its genome sequenced, its genetic blueprint broadens our understanding of photosynthesis in extreme environments and fills in one of the significant gaps in genomic databases of anoxygenic phototrophic bacteria. Insights from this genome and from future genomic studies of phototrophic extremophiles should help clarify the origin and evolution of photosynthesis and the transition from anoxygenic to oxygenic phototrophy.

## Figures and Tables

**Table 1:** Classification and general features of *Rhodoferax antarcticus* str. ANT.BR^T^ *.

Property	Term
Classification	Domain: *Bacteria*
	Phylum: *Proteobacteria*
	Class: *Betaproteobacteria*
	Order: *Burkholderiales*
	Family: *Comamonadaceae*
	Genus: *Rhodoferax*
	Species: *Rhodoferax antarcticus*
	Type strain: ANT.BR^T^ (ATCC 700587; DSMZ 24876)
Gram stain	Negative
Cell shape	Vibrio to spirillum
Motility	Highly motile
Endosporulation	Non-endospore forming
Temperature range	0–25 °C
Optimum temperature	15–18 °C
pH range; Optimum	6–8; 7
Carbon sources	Acetate, pyruvate, lactate, succinate, malate, fumarate, glucose, fructose, sucrose, citrate, aspartate
Habitat	Algal–bacterial microbial mat
Salinity	0%–2% NaCl (*w*/*v*)
Oxygen requirement	Facultative aerobe
Biotic relationship	Free-living
Pathogenicity	Non-pathogenic
Geographic location	Cape Royds, Ross Island, Antarctica
Sample collection	December, 1993
Latitude	77.55° S
Longitude	166.16° E
Altitude	20 m

* Data are adapted from Madigan et al. [3].

**Table 2:** Features of the *Rhodoferax antarcticus* str. ANT.BR^T^ genome.

Attribute	Value	% of Total
Genome size (bp)	4,007,881	100.0
Chromosome size (bp)	3,809,266	95.0
Plasmid size (bp)	198,615	5.0
DNA coding (bp)	3,564,951	88.9
Chromosome G + C content	59.1	–
Plasmid G + C content	48.4	–
Total genes	4324	100.0
Protein-encoding genes	4257	98.5
RNA genes	67	1.5
Pseudogenes (putative)	228	5.3
Genes with function prediction	2606	60.2
Genes with Pfam domains	3130	72.3
Genes with signal peptides	211	4.9
Genes with transmembrane helices	800	18.5
CRISPR repeats	5	0.1

**Table 3:** Functional role categories of *Rhodoferax antarcticus* str. ANT.BR^T^ genes.

Characteristic	Genes	% of Genome Content *
Energy and central intermediary metabolism	438	10.3
Amino acid biosynthesis	107	2.5
Transport and binding proteins	342	8.0
Cofactor and prosthetic group biosynthesis	206	4.8
DNA metabolism and nucleotide synthesis	252	5.9
Transcription	76	1.8
Protein synthesis, modification, and degradation	332	7.8
Regulatory functions and signal transduction	299	7.0
Cellular processes (division, chemotaxis, motility, toxin production and resistance, detoxification)	262	6.2
Fatty acid and phospholipid metabolism	71	1.7
Mobile and extrachromosomal element functions	159	3.7
Cell envelope	251	5.9
Proteins with family/domain assignments	407	9.6
Hypothetical proteins	754	17.7
Conserved hypothetical proteins	558	13.1

* Because some genes apply to more than one role category, this total exceeds 100%.
